# Erratum: Golding et al. Examining the Effect of Context, Beliefs, and Values on UK Farm Veterinarians’ Antimicrobial Prescribing: A Randomized Experimental Vignette and Cross-Sectional Survey. *Antibiotics* 2021, *10*, 445

**DOI:** 10.3390/antibiotics10060729

**Published:** 2021-06-17

**Authors:** Sarah E. Golding, Jane Ogden, Helen M. Higgins

**Affiliations:** 1 School of Psychology, Faculty of Health and Medical Sciences, Stag Hill Campus, University of Surrey, Guildford GU2 7XH, UK; j.ogden@surrey.ac.uk; 2 Institute of Infection, Veterinary and Ecological Sciences, University of Liverpool, Neston, Cheshire CH64 7TE, UK; H.Higgins@liverpool.ac.uk

The authors wish to make the following corrections to their published paper [1]. The hyperlink to the open dataset that accompanies this paper was incorrect in the original published Data Availability Statement. In the original publication the link was: ‘http://doi.org/10.5255/UKDA-SN-854821kdj’. The correct hyperlink is: ‘http://doi.org/10.5255/UKDA-SN-854821’.

The corrected Data Availability Statement should be: 

**Data Availability Statement:** Data available in a publicly accessible repository. The data presented in this study are openly available in UK Data Service at http://doi.org/10.5255/UKDA-SN-854821 (Data Collection title: Experimental vignette and cross-sectional survey with farm veterinarians, 2018). Demographic data have been removed from the publicly accessible file to ensure participant anonymity.

The authors would like to apologize for any inconvenience caused to the readers by these changes. The change does not affect the results or conclusions of the study.
