# Supplementary material for: Examining the Effect of Context, Beliefs, and Values on UK Farm Veterinarians’ Antimicrobial Prescribing: A Randomized Experimental Vignette and Cross-Sectional Survey
Source: Antibiotics (Basel). 2021 Apr 15;10(4):445. doi: 10.3390/antibiotics10040445 (PMC8071438; doi:10.3390/antibiotics10040445)
Supplement: Supplementary file 1 [file antibiotics-10-00445-s001.zip › antibiotics-1141296-supplementary.pdf]

Notes relating to assumptions for inferential tests performed

Impact of vignette on prescribing (H1a, H1b): The outcome variable was normally distributed; these hypotheses were tested using a one-way independent ANOVA (assumption of homogeneity of variances met; Levene's statistic (3,93) = 1.16, $p = 0.33$).

Vets' beliefs about groups' responsibility for causing and preventing AMR (H2a, H2b): To explore potential differences in beliefs, two Friedman's ANOVAs were conducted, as four of the twelve subscales measuring these beliefs were not normally distributed (cause beliefs: public/patients and pet owners; prevent beliefs: human medics and farmers).

Impact of beliefs on prescribing (H2c, H2d): These hypotheses were tested using two bootstrapped hierarchical multiple regressions; assumptions of homoscedasticity, linearity, independence of errors, normally distributed errors, and no multicollinearity were met for both regressions, but there were multivariate outliers which may have exerted undue influence. In both regressions, vignette condition was entered in block one, and beliefs about all groups either causing or preventing AMR were entered in block two.

Impact of values on prescribing (H3a, H3b, H3c, H3d): These hypotheses were tested using a bootstrapped hierarchical multiple regression; vignette condition was entered in block one and the four values subscales were entered in block two. All assumptions were met, and no multivariate outliers were identified.

Supplemental Table S1. Categorical Demographic Variables by Condition.

Measure	Response Option	Number of Participants per Condition (Percentage of Sample who Provided Response)			
		**Control (*n* = 22)**	**Economics (*n* = 26)**	**Farmer (*n* = 24)**	**Relationship (*n* = 25)**
Gender ($n = 92$) [1]	Female	9 (19.6)	12 (26.1)	14 (30.4)	11 (23.9)
	Male	13 (28.9)	10 (22.2)	9 (20.0)	13 (28.9)
	Other	0 (0.0)	0 (0.0)	0 (0.0)	0 (0.0)
	Prefer Not to Say	0 (0.0)	1 (100.0)	0 (0.0)	0 (0.0)
Ethnicity ($n = 92$) [2]	White [5]	21 (23.1)	23 (25.3)	23 (25.3)	24 (26.4)
	Black	0 (0.0)	0 (0.0)	0 (0.0)	0 (0.0)
	Asian	0 (0.0)	0 (0.0)	0 (0.0)	0 (0.0)
	Mixed	0 (0.0)	0 (0.0)	0 (0.0)	0 (0.0)
	Other	0 (0.0)	0 (0.0)	0 (0.0)	0 (0.0)
	Prefer Not to Say	1 (100.0)	0 (0.0)	0 (0.0)	0 (0.0)
Holds Postgraduate Veterinary Qualifications ($n = 91$) [3]	Yes [5]	8 (30.8)	5 (19.2)	7 (26.9)	6 (23.1)
	No [5]	14 (21.5)	17 (26.2)	16 (24.6)	18 (27.7)
Primary Region of Work ($n = 92$) [4]	Northern Ireland	0 (0.0)	0 (0.0)	0 (0.0)	1 (100.0)
	Scotland	0 (0.0)	3 (60.0)	0 (0.0)	2 (40.0)
	Wales	1 (20.0)	0 (0.0)	2 (40.0)	2 (40.0)
	North East England / Yorkshire & Humber [5]	3 (42.9)	1 (14.3)	2 (28.6)	1 (14.3)
	North West England [5]	4 (21.1)	6 (31.6)	3 (15.8)	6 (31.6)
	Midlands (East/West) [5]	1 (16.7)	1 (16.7)	4 (66.7)	0 (0.0)
	East of England	1 (25.0)	0 (0.0)	1 (25.0)	2 (50.0)
	South East England [5]	4 (44.4)	3 (33.3)	1 (11.1)	1 (11.1)
	South West England	5 (19.2)	4 (15.4)	9 (34.6)	8 (30.8)
	Other	1 (100.0)	0 (0.0)	0 (0.0)	0 (0.0)
	Multiple	2 (22.2)	5 (55.6)	1 (11.1)	1 (11.1)

[1] Test of diffference: Fisher's Exact Test = 4.99, $p = 0.55$. [2] Test of diffference: Fisher's Exact Test = 2.99, $p = 0.24$. [3] Test of difference: Pearson's $\chi^2 = 1.21$ (degrees of freedom = 3), $p = 0.76$. [4] Test of diffference: Fisher's Exact Test = 30.74, $p = 0.28$. [5] Percentages do not exactly total 100% due to rounding.

Supplemental Table S2. Continuous Demographic Variables, Values, and Beliefs by Condition.

Measure	Response Option	Mean (SD)	BCa 95% CI	Mean (SD)	BCa 95% CI	Mean (SD)	BCa 95% CI	Mean (SD)	BCa 95% CI
Age (*n* = 80) [1]	-	30.50 (16.00)		31.50 (8.00)		34.50 (28.00)		33.50 (17.00)	
Years Qualified (*n* = 92) [1]	-	7.50 (17.00)		8.00 (9.00)		11.00 (12.00)		9.50 (15.00)	
Time Spent Working with Animals (*n* = 97)	Dairy Cattle [1]	4.00 (1.00)		4.00 (0.25)		4.00 (0.75)		4.00 (0.00)	
	Beef Cattle [1]	4.00 (0.00)		4.00 (0.25)		4.00 (1.00)		4.00 (0.50)	
	Sheep [1]	4.00 (1.00)		4.00 (1.00)		4.00 (1.00)		4.00 (1.00)	
	Goats	2.77 (0.92)	[2.36, 3.14]	2.54 (0.86)	[2.21, 2.88]	2.92 (0.88)	[2.55, 3.26]	2.56 (0.92)	[2.17, 2.95]
	Pigs	2.14 (0.64)	[1.89, 2.40]	1.92 (0.74)	[1.65, 2.25]	2.17 (0.76)	[1.90, 2.44]	1.92 (0.70)	[1.67, 2.20]
	Poultry (Chickens)	2.18 (0.59)	[1.95, 2.40]	1.85 (0.79)	[1.57, 2.15]	2.04 (0.81)	[1.74, 2.36]	1.80 (0.65)	[1.52, 2.07]
	Poultry (Other) [1]	2.00 (1.00)		2.00 (1.00)		2.00 (1.00)		2.00 (1.00)	
	Other Farm Animals [1]	2.00 (1.00)		2.00 (0.00)		2.00 (1.00)		2.00 (1.50)	
	Horses [1]	1.50 (1.00)		1.50 (1.00)		1.00 (1.00)		1.00 (1.00)	
	Small Animals [1]	2.00 (1.25)		1.00 (1.25)		1.00 (1.00)		1.00 (1.00)	
Values (*n* = 92)	Hedonic	6.48 (0.98)	[6.08, 6.88]	6.24 (1.25)	[5.71, 6.73]	6.41 (1.34)	[5.87, 6.91]	6.40 (1.70)	[5.71, 7.00]
	Egoistic	4.96 (1.28)	[4.45, 5.51]	4.25 (0.97)	[3.86, 4.60]	4.96 (1.23)	[4.41, 5.42]	4.35 (1.10)	[3.92, 4.75]
	Altruistic	6.95 (1.17)	[6.50, 7.37]	6.44 (1.54)	[5.79, 7.05]	6.86 (1.34)	[6.29, 7.26]	6.82 (1.32)	[6.27, 7.26]
	Biospheric	6.13 (1.14)	[5.63, 6.65]	6.33 (1.50)	[5.67, 6.95]	6.57 (1.31)	[6.00, 7.09]	6.28 (1.52)	[5.62, 6.90]
Responsibility for Causing AMR (*n* = 89)	Human Medics	3.79 (0.58)	[3.52, 4.09]	3.87 (0.65)	[3.62, 4.10]	3.74 (0.82)	[3.46, 4.01]	3.70 (0.69)	[3.44, 3.95]
	Public/Patients	3.82 (0.59)	[3.58, 4.05]	3.75 (0.81)	[3.43, 4.07]	3.47 (0.81)	[3.15, 3.83]	3.84 (0.75)	[3.51, 4.15]
	Companion Animal Vets	3.07 (0.59)	[2.79, 3.32]	3.15 (0.88)	[2.81, 3.48]	3.16 (0.80)	[2.86, 3.52]	2.85 (0.62)	[2.60, 3.10]
	Pet Owners	3.21 (0.76)	[2.88, 3.55]	3.25 (0.91)	[2.87, 3.62]	3.11 (0.89)	[2.76, 3.50]	3.16 (0.75)	[2.88, 3.48]
	Farm Animal Vets	3.20 (0.72)	[2.88, 3.57]	3.25 (0.74)	[2.95, 3.53]	3.38 (0.60)	[3.14, 3.59]	2.98 (0.71)	[2.69, 3.27]
	Farmers	3.61 (0.76)	[3.24, 3.96]	3.50 (0.78)	[3.21, 3.81]	3.76 (0.70)	[3.47, 4.05]	3.60 (0.68)	[3.36, 3.84]
Responsibility for Preventing AMR (*n* = 88)	Human Medics	4.32 (0.61)	[4.00, 4.61]	4.38 (0.50)	[4.17, 4.57]	4.18 (0.66)	[3.88, 4.45]	4.24 (0.72)	[3.93, 4.50]
	Public/Patients	4.02 (0.63)	[3.75, 4.29]	4.18 (0.56)	[3.94, 4.40]	3.95 (0.64)	[3.70, 4.19]	4.15 (0.72)	[3.87, 4.44]

Companion Animal Vets	3.54 (0.77)	[3.22, 3.85]	3.81 (0.74)	[3.51, 4.11]	3.58 (0.73)	[3.24, 3.88]	3.60 (0.76)	[3.31, 3.89]
Pet Owners	4.07 (0.66)	[3.77, 4.33]	3.85 (0.68)	[3.57, 4.11]	3.86 (0.75)	[3.56, 4.18]	3.96 (0.80)	[3.64, 4.26]
Farm Animal Vets	4.06 (0.56)	[3.82, 4.30]	4.07 (0.58)	[3.84, 4.31]	4.13 (0.54)	[3.89, 4.35]	4.00 (0.71)	[3.70, 4.28]
Farmers	4.35 (0.61)	[4.05, 4.64]	4.11 (0.54)	[3.88, 4.39]	4.19 (0.68)	[3.89, 4.47]	4.19 (0.77)	[3.88, 4.48]

[1] Median and interquartile range reported for non-parametric variables. [2] BCa 95% CI = 95% bias-corrected and accelerated bootstrap confidence intervals. Bootstrap results are based on 1,000 bootstrap samples.

Supplemental Table S3. Significance Checks for Baseline Differences for Continuous Demographic Variables.

Measure	Response Option	ANOVA F-ratio (df)	Kruskal-Wallis H (df)	p
Age (n = 80)	-		1.60 (3)	0.66
Years Qualified (n = 92)	-		1.50 (3)	0.68
Time Spent Working with Animals (n = 97)	Dairy Cattle		3.63 (3)	0.31
	Beef Cattle		1.47 (3)	0.69
	Sheep		0.25 (3)	0.97
	Goats	1.00 (3,93)		0.40
	Pigs	0.84 (3,93)		0.47
	Poultry (Chickens)	1.45 (3,93)		0.24
	Poultry (Other)		6.01 (3)	0.11
	Other Farm Animals		5.70 (3)	0.13
	Horses		3.33 (3)	0.34
	Small Animals		1.87 (3)	0.60
Values (n = 92)	Hedonic	0.18 (3, 48.79) [1]		0.91
	Egoistic	2.54 (3,88)		0.062
	Altruistic	0.64 (3,88)		0.59
	Biospheric	0.38 (3,88)		0.77
Responsibility for Causing AMR (n = 89)	Human Medics	0.26 (3,85)		0.85
	Public/Patients	1.17 (3,85)		0.33
	Companion Animal Vets	1.03 (3, 46.21) [1]		0.39
	Pet Owners	0.12 (3,85)		0.95
	Farm Animal Vets	1.33 (3,85)		0.27
	Farmers	0.49 (3,85)		0.69
Responsibility for Preventing AMR (n = 88)	Human Medics	0.42 (3,84)		0.74
	Public/Patients	0.26 (3,84)		0.60
	Companion Animal Vets	0.31 (3,84)		0.65
	Pet Owners	0.40 (3,84)		0.76
	Farm Animal Vets	0.16 (3,84)		0.92
	Farmers	0.47 (3,84)		0.70

[1] Welch's F reported as homogeneity of variances not assumed.

Supplemental Table S4. Hierarchical Regression Model Summaries for Demographics as Predictors of Prescribing.

Measure	*F* (df)	*p*	R^2	*F* (df)	*p*	ΔR^2	*p*
Age (*n* = 80)	1.20 (3,76)	0.31	0.045	0.93 (4,75)	0.45	0.002	0.72
Gender (*n* = 92)	1.50 (3,88)	0.22	0.049	1.00 (5,86)	0.42	0.006	0.75
Years Qualified (*n* = 92)	1.20 (3,88)	0.22	0.049	1.36 (4,87)	0.25	0.010	0.33
Holds Postgraduate Qualifications (*n* = 91)	1.44 (3,87)	0.24	0.047	1.20 (4,86)	0.32	0.006	0.48
Time Spent Working with Animals (*n* = 97)	1.64 (3,93)	0.19	0.050	1.36 (13,83)	0.20	0.126	0.26
Primary Region of Work (*n* = 92)	1.50 (3,88)	0.22	0.049	1.11 (13,78)	0.36	0.108	0.45

Supplemental Table S5. Pairwise Comparisons for Beliefs about Groups' Responsibilities for Causing and Preventing AMR.

Measure	Comparison	Mean Difference	Std. Test Statistic	*Adj. p*	*r*
Responsibility for Causing AMR (*n* = 89)	Farm Vets v. Farmers	-0.42	5.79	< 0.001	0.43
	Farm Vets v. Human Medics	-0.58	5.85	< 0.001	0.44
	Farm Vets v. Public/Patients	-0.53	5.95	< 0.001	0.45
	Farm Vets v. Companion Animal Vets	0.14	1.42	1.00	0.11
	Farm Vets v. Pet Owners	0.01	0.06	0.95	0.004
	Farmers v. Human Medics	-0.15	0.06	0.95	0.004
	Farmers v. Public/Patients	-0.10	0.16	0.87	0.01
	Farmers v. Companion Animal Vets	0.57	7.21	< 0.001	0.54
	Farmers v. Pet Owners	0.44	5.73	< 0.001	0.43
	Human Medics v. Public/Patients	0.05	0.10	0.92	0.007
	Human Medics v. Companion Animal Vets	0.72	7.27	< 0.001	0.55
	Human Medics v. Pet Owners	0.59	5.79	< 0.001	0.43
	Public/Patients v. Companion Animal Vets	0.67	7.37	< 0.001	0.55
	Public/Patients v. Pet Owners	0.54	5.89	< 0.001	0.44
	Companion Animal Vets v. Pet Owners	-0.13	1.48	1.00	0.11
Responsibility for Preventing AMR (*n* = 88)	Farm Vets v. Farmers	-0.15	2.82	0.072	0.21
	Farm Vets v. Human Medics	-0.22	3.08	0.031	0.23
	Farm Vets v. Public/Patients	-0.02	0.26	1.00	0.02
	Farm Vets v. Companion Animal Vets	0.43	4.94	< 0.001	0.37
	Farm Vets v. Pet Owners	0.13	1.47	1.00	0.11
	Farmers v. Human Medics	-0.07	0.26	1.00	0.02
	Farmers v. Public/Patients	0.13	2.56	0.16	0.19
	Farmers v. Companion Animal Vets	0.58	7.76	< 0.001	0.58
	Farmers v. Pet Owners	0.28	4.29	< 0.001	0.32
	Human Medics v. Public/Patients	0.20	2.82	0.072	0.21
	Human Medics v. Companion Animal Vets	0.65	8.02	< 0.001	0.60
	Human Medics v. Pet Owners	0.35	4.55	< 0.001	0.34
	Public/Patients v. Companion Animal Vets	0.45	5.20	< 0.001	0.39
	Public/Patients v. Pet Owners	0.15	1.73	1.00	0.13
	Companion Animal Vets v. Pet Owners	-0.30	3.47	0.008	0.26
